# Assessment of the caudate nucleus and its relation to route learning in both congenital and late blind individuals

**DOI:** 10.1186/1471-2202-14-113

**Published:** 2013-10-04

**Authors:** Patrice Voss, Madeleine Fortin, Vincent Corbo, Jens C Pruessner, Franco Lepore

**Affiliations:** 1 Montreal Neurological Institute, McGill University, Montréal, Canada; 2 Centre de Recherche en Neuropsychologie et Cognition (CERNEC), Université de Montréal, Montréal, Canada; 3 Douglas Hospital Research Center, McGill University, Montréal, Québec, Canada

**Keywords:** Blindness, Caudate nucleus, Spatial navigation, Volumetric MRI

## Abstract

**Background:**

In the absence of visual input, the question arises as to how complex spatial abilities develop and how the brain adapts to the absence of this modality. As such, the aim of the current study was to investigate the relationship between visual status and an important brain structure with a well established role in spatial cognition and navigation, the caudate nucleus. We conducted a volumetric analysis of the caudate nucleus in congenitally and late blind individuals, as well as in matched sighted control subjects.

**Results:**

No differences in the volume of the structure were found either between congenitally blind (CB) and matched sighted controls or between late blind (LB) and matched sighted controls. Moreover, contrary to what was expected, no significant correlation was found between caudate volume and performance in a spatial navigation task. Finally, consistent with previously published reports, the volume of the caudate nucleus was found to be negatively correlated with age in the sighted; however such correlations were not significant in the blind groups.

**Conclusion:**

Although there were no group differences, the absence of an age-volume correlation in the blind suggests that visual deprivation may still have an effect on the developmental changes that occur in the caudate nucleus.

## Background

Spatial cognition and the ability to properly navigate in one’s environment are believed to result from the contribution of several subcortical structures such as the hippocampus (HC) and the caudate nucleus (CN). This is well documented in rats, for instance, as place learning involves two different memory systems subserved by the HC and the dorsal striatum (particularly the caudate nucleus) [1-3]. In the early phases of learning, the HC is involved in the rapid acquisition of spatial information, allowing for rats to reach a target from any starting position [1]. The dorsal striatum is involved in a slower learning process [4] that relies on rewarded stimulus–response (S-R) behaviour [3,5], i.e. gradually learning particular body turns in response to stimuli, which allow the rats to reach a target location from one starting point [6]. A similar segregation has been observed in humans. Functional Magnetic Resonance Imaging (fMRI) studies have shown that tasks requiring spatial representations preferentially activate the HC, while tasks not requiring a particular `spatial strategy activate mainly the CN [7,8]. Moreover, gray matter density in these structures is found to correlate with specific navigational strategies [9]; subjects who were qualified as “spatial learners” had significantly more grey matter in the HC and less in the CN compared to “response learners”.

Given the importance of vision and visual cues for spatial navigation and wayfinding, the absence of the visual modality raises questions on not only the ability to navigate in one’s environment without vision but also on the anatomical and functional consequences to the brain structures involved in such learning. Despite the absence of visual inputs, blind individuals are nonetheless able to properly orient themselves and navigate in space [10,11]. To date, three studies have examined the effects of visual loss on the structural integrity of the HC [11-13]. While initial results may have seemed at odds with each other, with Fortin et al. [11] finding an increase in volume in the anterior portion of the HC and Chebat et al. [12] finding a decrease in volume in the posterior portion, a subsequent study by Lepore et al. [13] actually confirmed both sets of findings, suggesting that following blindness there is a shift of the neuronal population towards the anterior portions of the hippocampus compared to sighted individuals. Importantly, however, little is known about the consequences of visual loss on the structural anatomy of the CN. Interestingly, in our previous study [11], where both blind and sighted subjects were asked to learn new paths in a human-size labyrinth (i.e. route learning task), most subjects anecdotally reported using a ‘response learners’ strategy where they attempted to sequentially recall the series of right and left turns that were required to properly follow the taught route. Indeed, previous work has not only shown the blind to possess superior serial memory for sequences [14], but that they also construct mental representations of paths via serial memorization of segmented inputs from each location along the path [15]. Moreover, crucial to our hypothesis formulation, recent data has in fact shown that route learning abilities in young and older adults are positively correlated to CN volume [16]. Therefore, given the above mentioned findings, we hypothesized here that blind individuals would not only possess larger CN relative to their sighted counterparts, but also that the volume would be significantly related to previously obtained behavioral measures in the blind [11]. To address this, using the MRI scans previously obtained in the aforementioned hippocampus volumetric study [11], we performed volume measurements of the CN in congenitally blind, late blind and matched sighted controls. We furthermore wanted to assess whether known associations with the caudate volume would also hold true in blindness. For instance, in normally-sighted individuals, the caudate volume is known to decrease with age [17,18], and there are conflicting reports on how the caudate and hippocampal volumes covary with one another [9,19,20]. The latter investigation is especially pertinent to the present study, given the previously established relationship between hippocampal volume and route learning performance.

## Methods

### Subjects

Thirty participants, with no history of neurological, cognitive or sensory-motor deficits, other than blindness in the case of the blind participants, took part in the study and were divided into four groups (see Table 1 for demographic data on the blind participants). The first group (n = 8) consisted of congenitally blind (CB) individuals (age: 31.1 ± 9.5 y; gender: 6 m/2 f; 7 right-handers), while the second group (n = 8) were sighted (SCB) participants (age: 31.4 ± 9.4 y; gender: 6 m/2 f; 7 right-handers) paired for age, gender, education and laterality, as assessed by the *Edinburgh Handedness Inventory*[21]. The third group (n = 7) consisted of late-blind (LB) individuals (age: 39.9 ± 12.7 y; gender: 4 m/3 f; 6 right-handers) who all lost their vision after 14 years of age (age of onset: 21.28 ± 6.55 y). The last group (n = 7) were sighted participants (SLB) matched with the LB individuals (age: 40.3 ± 13.4 y; gender: 4 m/3 f; 6 right-handers). The research protocol was approved by the ethics committees of the Center for Interdisciplinary Research in Rehabilitation, which coordinates in the Province of Quebec research with blind participants and is sponsored by the Institut Nazareth & Louis Braille, by the Research Center of the Institut Universitaire de Gériatrie de Montréal, where the testing was carried out, by the Centre Hospitalier de l’Université de Montréal (CHUM), where the MRI scans were collected, and by the Université de Montréal, where the project originated. All participants provided written informed consent prior to testing.

**Table 1:** Subject demographic information

**Subjects**	**Age**	**Sexe**	**Onset**	**Duration**	**LP**	**Causes of blindness**
EB1	19	M	0	19	Yes	Leber’s congenital amaurosis
EB2	22	M	0	22	No	Leber’s congenital amaurosis
EB3	46	F	0	46	Yes	Rubella
EB4	35	M	0	35	No	Retinal detachment
EB5	39	M	0	39	No	Retinitis pigmentosa
EB6	31	M	0	31	No	Congenital glaucoma
EB7	35	M	0	35	Yes	Leber’s congenital amaurosis
EB8	22	F	0	22	No	Retinal detachment
LB1	24	F	17	7	Yes	Glaucoma
LB2	40	M	21	19	No	Unknown
LB3	47	F	27	20	Yes	Ischemic retinopathy
LB4	22	M	16	6	Yes	Retinitis pigmentosa
LB5	57	M	33	24	Yes	Medical accident (retina damage)
LB6	42	M	15	27	No	Congenital cataracts and glaucoma
LB7	47	F	20	27	No	Glaucoma and aniridia

The ‘Onset’ column refers to the age at which the subjects lost their sight. The ‘Duration’ column refers to the number of years that the subjects have been blind. The ‘LP’ column indicates whether subjects still had any residual light perception.

### Image acquisition and analysis

For each participant, MR images were previously acquired (see [11]) on a Siemens 1.5 Tesla Magnetom Vision MRI scanner (Siemens, Erlangen, Germany) at the Notre-Dame Hospital (CHUM). All images were acquired in high resolution (1 × 1 × 1 mm, T1-weighted 3D) with a sagittally oriented echo sequence (TR: 1100; TE:4.38; flip angle of 15; 256 × 256 matrix and FOV:250).

**Figure 1:**
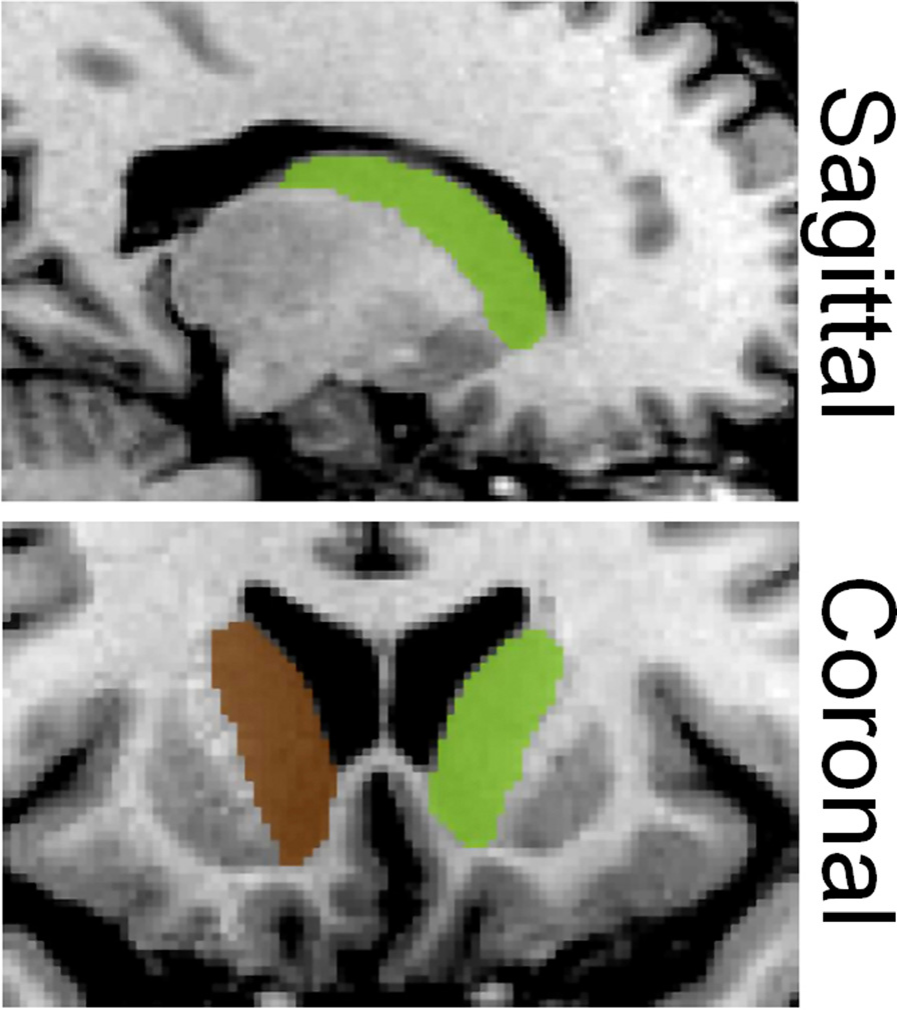
**Segmented caudate nucleus.** Segmented caudate nuclei in both the sagittal (top panel) and coronal plane (bottom panel; brown = left caudate; green = right caudate).

The raw structural files were first corrected for non-uniformities [22], and then registered into standard stereotaxic space based on the MNI 152 template [23]. This transformation results in an alignment along the AC-PC axis and accounts for individual differences in global brain size and shape. The images were then classified into different maps of gray matter, white matter, and cerebrospinal fluid (CSF) [24]. This procedure included the removal of all extracranial tissue, dura and the cerebellum. The resulting brain images were subsequently non-linearly registered and used to compute labels for the CN with the software ANIMAL [25], using each subject’s classified maps. ANIMAL generates labels by multiplying the voxel values of the templates of the CN with each voxel’s value across the individual grey matter maps (values of 1 for grey matter, 0 for either white matter or CSF). Manual corrections and volume assessments were performed by a trained rater (PV) to correct for gray-white matter boundaries and partial volume effects using the software package DISPLAY, developed at the Montreal Neurological Institute [26,27]. DISPLAY allows simultaneous viewing and navigating of brain volumes in coronal, sagittal, and horizontal orientations in 1mm slice intervals. Figure 1 illustrates the resulting segemented caudate nucleus in one of the subjects.

Following the manual corrections, the reliability of the measurements was ascertained by obtaining estimates of both the intra-rater reliability and inter-rater reliability. The intra-rater intra-class reliability coefficients were 0.971 (left CN) and 0.988 (right CN), and were obtained by the original rater (PV) re-measuring the CN volume in four randomly selected MRI scans, with at least two months elapsing between consecutive measurements. The inter-rater intra-class reliability coefficients were 0.926 (left CN) and 0.942 (right CN), and were obtained by two raters (PV and MF) measuring caudate volumes independently in eight randomly selected MRI scans.

### Behavioral route learning task

The task is described in greater detail Fortin et al. [11]. Briefly, the route learning task was performed as follows: subjects were asked to memorize a traveled route within a human-size labyrinth, guided by an experimenter, and then had to follow the same path alone on five subsequent trials while trying to make as few errors as possible; when mistakes were made, subjects were instructed to stop and were repositioned by the experimenter in the correct direction. Sighted subjects performed the task blindfolded. Four different routes were tested of increasing difficulty: with six, eight, ten and twelve decision points to memorize.

## Results

### Volumetric analyses

**Figure 2:**
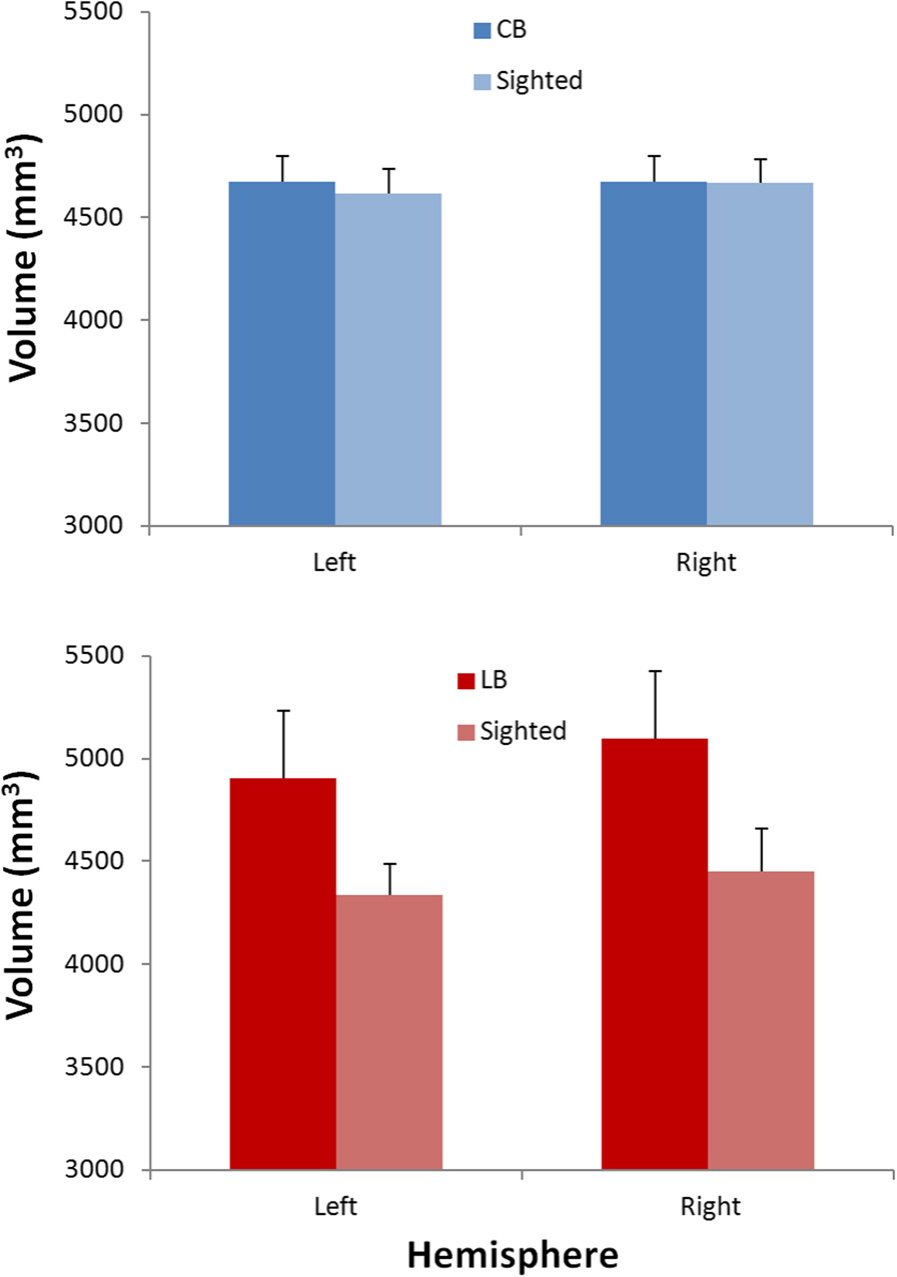
**Group effect on caudate volume.** Illustrated here are the caudate volumes for each hemisphere for all four groups. The top panel contrasts the CB and the SCB, while the bottom panel contrasts the LB and the SLB. Error bars represent the standard error.

Separate analyses were performed for both blind groups since they were not matched with one another. A first two (group: CB and SCB) by two (hemisphere) repeated measures ANOVA revealed that there was no significant group effect (F = 0.034; p = 0.856), no effect of hemisphere (F = 0.241; p = 0.631), nor a significant group x hemisphere interaction (F = 0.314; p = 0.584). A second two (group: LB and SLB) by two (hemisphere) repeated measures ANOVA did reveal a significant hemisphere effect (F = 6.995; p = 0.021) as the right caudate was found to be larger than the left one (see Figure 2); however the group effect (F = 2.659; p = 0.129) and the group x hemisphere interaction (F = 0.465; p = 0.508) were non-significant.

### Correlational analyses

We correlated caudate volumetric measurements with several other measures to address aforementioned hypotheses. Both sighted control groups were pooled together, as there is no a priori reason to not consider them along a continuum of individuals with normal sensory experience. Regression analyses involving the blind groups were performed with both groups pooled together, as well as separately since both groups have differential experience with the visual modality.

We first ascertained whether or not caudate volume would be predictive of performance on a spatial navigation task. The task is briefly explained above, and entailed the learning of 4 different routes of increasing difficulty within a human size labyrinth. Previously, the size of the right hippocampus was shown to be predictive of performance. For a more detailed description of the task and of the findings, please see Fortin et al. [11]. As previously done, we chose to use the performance of each subject (number of errors) on the most difficult route as regressors in correlational analyses with caudate volume. However, contrary to what was observed with the hippocampus, no such correlation between performance and caudate volume was observed when correlating both measures across all participants [lCN (r = 0.011; p = 0.955); rCN (r = −0.144; p = 0.447)].

We also investigated the relationship between the total caudate volume and the total hippocampal volume previously measured in the same subjects (Fortin et al., [11]). We found no significant correlation between the volumes of both structures in any of the groups [CB (r = −0.263; p = 0.529), LB (r = 0.078; p = 0.869), Sighted (r = 0.242; p = 0.385)].

**Figure 3:**
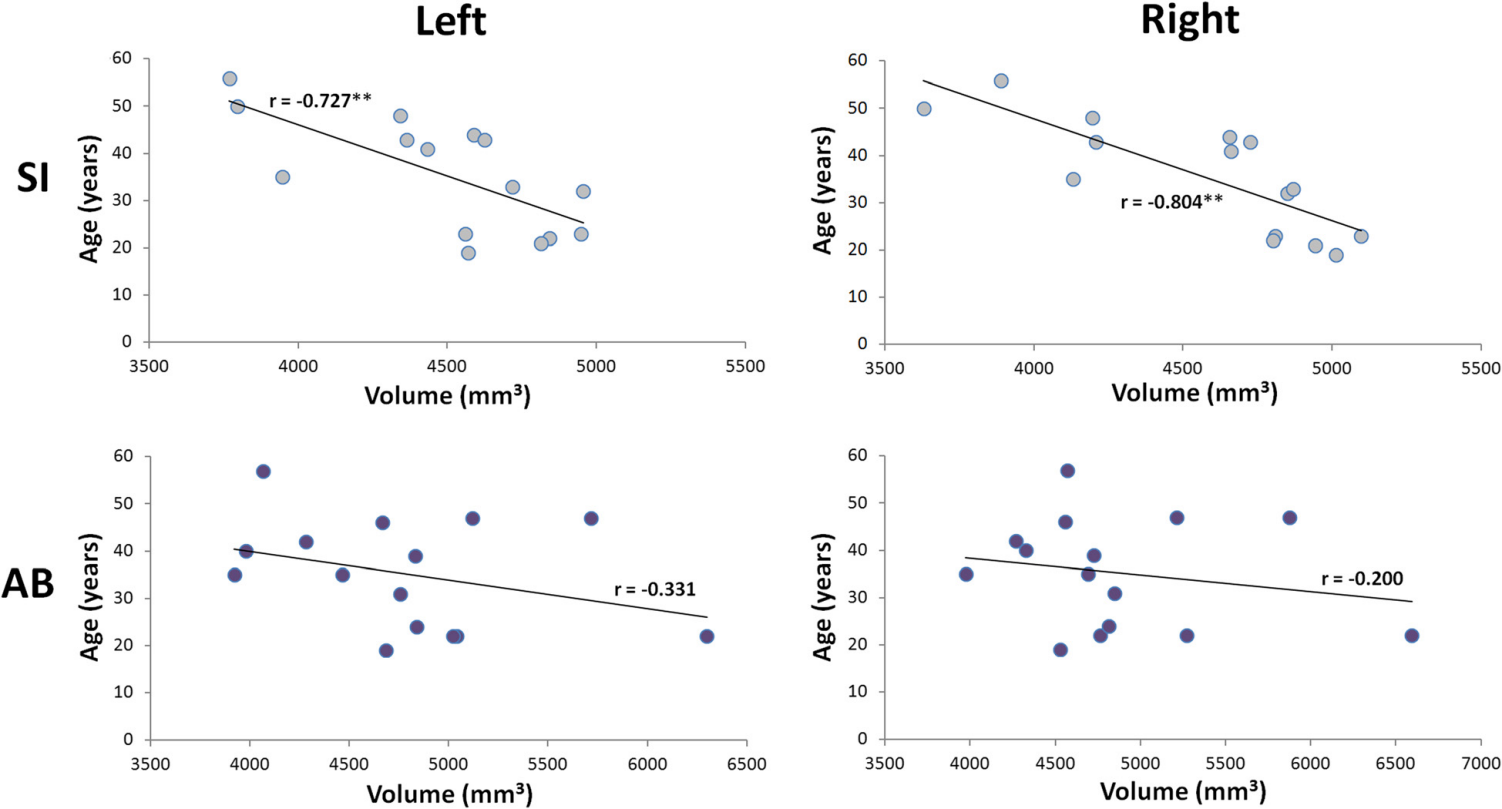
**Effect of age on caudate volume.** Illustrated here are the correlations between the age of the subjects and the caudate nucleus volume in the sighted (SI – top panel) and blind (AB: all blind subjects combined – bottom panel) subjects respectively. While there is a significant negative correlation between both measures in the sighted, indicating an age-related decline in caudate volume, the correlation in the blind (both CB and LB combined) is much weaker and did not reach statistical significance.

Lastly, we examined whether age at testing was related to CN volume. As seen in Figure 3, caudate volume was inversely correlated with age for sighted subjects [lCN (r = −0.737; p = 0.002); rCN (r = −0.804; p < 0.001)]. However, this relationship was much weaker and did not reach statistical significance either for the CB [lCN (r = −0.388; p = 0.342); rCN (r = −0.353; p = 0.391)] or for the LB [lCN (r = −0.485; p = 0.367); rCN (r = −0.405; p = 0.367)]. When pooling both blind groups together, given the similar correlation coefficients, the correlation coefficients became smaller and remained non-significant [lCN (r = −0.331; p = 0.229); rCN (r = −0.200; p = 0.475)]. A direct comparison of the correlation coefficients between the sighted and the blind, using Fisher’s z-transform, shows that they are significantly different from one another for the right caudate (z = 2.20, p = 0.026); the difference for the left caudate failed to reach statistical significance however (z = 1.47, p = 0.142).

## Discussion

The primary goals of the present study were: 1) to address whether or not the CN volume is affected in blindness, 2) to ascertain if it plays a role in the superior wayfinding abilities of blind individuals. Importantly, we also investigated the role played by the age of blindness onset on the CN (by separately comparing CB and LB to groups of sighted controls). Lastly, we also investigated whether blindness would alter associations the caudate volume has with hippocampal volume (disputed) and age.

### Effect of blindness

The CN volume was not significantly different in the CB compared to matched sighted controls (SCB). However, while Figure 2 seems to indicate that the CN is larger in the LB compared to the SLB, this difference did not reach statistical significance, possibly due to the small subject sample and high variability. The latter contrast also revealed a significant effect of hemisphere, with the right caudate being larger than the left for each group. This hemispheric asymmetry is consistent with previous research showing that the right caudate tends to be larger than the left caudate, irrespective of gender and laterality [28,29]. It is however unclear to us why this asymmetry was not found for the CB-SCB contrast. Since our LB and SLB groups are older, perhaps the CN asymmetry is exacerbated with aging. Such a hypothesis is however speculative and remains to be further explored.

We also chose to compare the CN measurements with those of previously obtained hippocampal measurements [11] in an attempt to address inconsistencies in the literature. Here we show that both measures are uncorrelated in all groups. This is in marked contrast to two separate lines of findings. The first stems from a previously mentioned study investigating the neural correlates of spatial navigation and showed that the size of both structures are negatively correlated with one another [9]. This inverse correlation is consistent with the authors’ findings of the hippocampus being larger in ‘spatial’ learners and of the CN being larger in ‘response’ learners. The second line of evidence stems from work investigating the effect of aging on cortical and subcortical structures, which has shown the volume of both the hippocampus and the CN to correlate with one another [19,20]. However in the latter studies, the correlation coefficients were modest (ranging between 0.24 and 0.4) and likely reached statistical significance due to the large sample sizes (> 65). Interestingly, the correlation found here in the sighted was also of 0.24 (though with a much smaller sample size), indicating a certain level of agreement between both datasets. It remains nonetheless difficult to reconcile our findings with those of Bohbot et al. [9], where a negative correlation was found. Consequently, the currently available data does not paint a very clear picture of the relationship between caudate and hippocampal volume. As such, further investigation will be required to elucidate the nature of the volumetric relationship, should one actually exist.

The present finding of an age-related decline of caudate volume in the sighted subjects was expected and is consistent with previous reports [17,18]. Surprisingly however, no such relationship was found in the blind. Furthermore, the strength of the age – caudate volume relationship was shown to be significantly different between the blind and the sighted for the right caudate, suggesting that blindness does perhaps affect the caudate volume in a more subtle manner than anticipated. The absence of a significant relationship in the blind appears to, at least partly, result from the fact that the CN volume in the older LB individuals isn’t reduced compared to the CB (see Figure 2). It would therefore appear that the loss of sight after puberty alters the normal developmental time-course of the age-related decline in caudate volume. The absence of an age-volume relationship in the blind could also possibly result from differential visual inputs into the tail of the CN via corticostriatal connections with extrastriate visual areas [30,31].

Taken together, the present findings indicate that the CN is not a beneficiary of compensatory plastic changes in blind individuals, as is the hippocampus [11,13] and visually-deafferented cortical areas. Indeed, there are numerous published reports showing that occipital brain function (see [32]) and occipital neuroanatomical changes [33] appear to underlie superior abilities in a wide-range of non-visual perceptual and cognitive domains. The present null finding might be due to the CN’s heterogeneous functionality which is largely motor-learning related, and for which there is little evidence of measured enhancements in the blind population. In contrast, the hippocampus’ primary functions are related to memory and spatial encoding, both for which the blind have been shown to develop superior abilities in [10,11,32]. The visually deafferented occipital cortex however, appears to underlie a variety of enhanced perceptual and cognitive skills in the blind. As such, given that route learning is but one of many functions subtended by the CN, it is possible that it is insufficient to drive substantial compensatory changes following visual deprivation.

### Relation to task performance

Given the nature of the spatial navigation task, where a prominent strategy for success was to sequentially recall the series of right and left, we had hypothesized that the CN would be a likely structure to be called upon, and that its volume would be predictive of performance as previous studies have shown [9]. Unexpectedly however, the CN volume was not at all predictive of performance. This finding was of course unexpected given the nature of the task and the known association between the type of task used and grey matter volume in the CN. Importantly though, it is worth pointing out that volume does not always correlate with performance in spatial navigation tasks, but rather correlates with strategies as previously underlined [9]. This was highlighted in a recent study where the hippocampal volume was found to correlate only with the use of spatial memory strategies, and not with performance on a navigation task [34]. Consequently, the absence of a correlation might simply reflect the use of differential strategies across our subject sample when performing the route learning task. However, without quantitative data on the strategies used by the participants, it is unfortunately difficult to explain the lack of a correlation with more certainty. Future work should take care in assessing the strategies used by the participants to address these unanswered questions.

## Conclusions

The present data suggest that compensatory plasticity in blindness does not extend to sub-cortical structures in the striatum, as the CN was found to be equal in volume between sighted and blind subjects (although a statistically non-significant increase was observed in the LB relative to sighted controls), and did not correlate with performance on of the route learning task. Evidently, further investigations are required in order to better understand the role visual deprivation plays in shaping the neuroanatomy of the CN, and how this interplays with route learning abilities.

## Competing interests

The authors declare that they have no competing interests.

## Authors’ contributions

Conception and design: PV, MF, JCP, FL. Data collection: PV, MF. Data analysis and interpretation: PV, VC, FL. Drafting of manuscript: PV. Revising and editing of manuscript: MF, VC, JCP, FL. All authors read and approved the final manuscript.

## References

[B1] O’Keefe J Nadel L The hippocampus as a cognitive Map 1978 Oxford: Clarendon

[B2] McDonald RJ White NM Parallel information processing in the water-maze: evidence for independent memory systems involving dorsal striatum and hippocampus Behav Neural Biol 1994 109 579 593 10.1016/s0163-1047(05)80009-3 8067981

[B3] White NM McDonald RJ Multiple parallel memory systems in the brain of the rat Neurobiol Learn Mem 2002 77 125 184 10.1006/nlme.2001.4008 11848717

[B4] Packard MG McGaugh JL Inactivation of hippocampus or caudate nucleus with lidocaine differentially affects expression of place and response learning Neurobiol Learn Mem 1996 65 65 72 10.1006/nlme.1996.0007 8673408

[B5] Packard MG Knowlton BJ Learning and memory functions of the basal ganglia Annu Rev Neurosci 2002 25 563 593 10.1146/annurev.neuro.25.112701.142937 12052921

[B6] Eichenbaum H Stewart C Morris HG Hippocampal representation in place learning J Neurosci 1990 10 3531 3542 2230943 10.1523/JNEUROSCI.10-11-03531.1990 PMC6570096

[B7] Hartley T Maguire EA Spiers HJ Burgess N The well-worn route and the path less travelled: distinct neural bases of route following and wayfinding in humans Neuron 2003 37 877 888 10.1016/S0896-6273(03)00095-3 12628177

[B8] Iaria G Petrides M Dagher A Pike B Bohbot VD Cognitive strategies dependent on the hippocampus and caudate nucleus in human navigation: variability and change with practice J Neurosci 2003 23 5945 5952 12843299 10.1523/JNEUROSCI.23-13-05945.2003 PMC6741255

[B9] Bohbot VD Lerch J Thorndycraft B Iaria G Zijdenbos AP Gray matter differences correlate with spontaneous strategies in a human virtual navigation task J Neurosci 2007 27 10078 10083 10.1523/JNEUROSCI.1763-07.2007 17881514 PMC6672675

[B10] Tinti C Adenzato M Tamietto M Cornoldi C Visual experience is not necessary for efficient survey spatial cognition: evidence from blindness Q J Exp Psychol 2006 59 1306 1328 10.1080/17470210500214275 16769626

[B11] Fortin M Voss P Lord C Lassonde M Pruessner J Saint-Amour D Rainville C Lepore F Wayfinding in the blind: larger hippocampal volume and supranormal spatial navigation Brain 2008 131 2995 3005 10.1093/brain/awn250 18854327

[B12] Chebat DR Chen JK Schneider F Ptito A Kupers R Ptito M Alterations in right posterior hippocampus in early blind individuals NeuroReport 2007 18 329 333 10.1097/WNR.0b013e32802b70f8 17435597

[B13] Lepore N Shi Y Lepore F Fortin M Voss P Chou Y Lord C Lassonde M Dinov I Toga AW Thompson PM Patterns of hippocampal shape and volume changes in blind subjects Neuroimage 2009 46 949 957 10.1016/j.neuroimage.2009.01.071 19285559 PMC2736880

[B14] Raz N Striem E Pundak G Orlov T Zohary E Superior serial memory in the blind: a case of cognitive compensatory adjustment Curr Biol 2007 17 1129 1133 10.1016/j.cub.2007.05.060 17583507

[B15] Iverson JM How to get to the cafeteria: gesture and speech in blind and sighted children’s spatial descriptions Dev Psychol 1999 35 1131 1142 10.1037//0012-1649.35.4.1132 10442881

[B16] Head D Isom M Age effects on wayfinding and route learning skills Behav Brain Res 2010 209 49 58 10.1016/j.bbr.2010.01.012 20085784

[B17] Hasan KM Halphen C Kamali A Nelson FM Wolinsky JS Narayana PA Caudate nuclei volume, diffusion tensor metrics, and T(2) relaxation in healthy adults and relapsing-remitting multiple sclerosis patients: implications for understanding gray matter degeneration J Magn Reson Imaging 2009 29 70 77 10.1002/jmri.21648 19097116 PMC2731422

[B18] Walhovd KB Fjell AM Reinvang I Lundervold A Dale AM Eilertsen DE Quinn BT Salat D Makris N Fischl B Effects of age on volumes of cortex, white matter and subcortical structures Neurobiol Aging 2005 26 1260 1270 10.1016/j.neurobiolaging.2005.05.020 16005549

[B19] Raz N Williamson A Gunning-Dixon F Head D Acker JD Neuroanatomical and cognitive correlates of adult age differences in acquisition of a perceptual-motor skill Microsc Res Tech 2000 51 85 93 10.1002/1097-0029(20001001)51:1<85::AID-JEMT9>3.0.CO;2-0 11002356

[B20] Raz N Lindenberger U Rodrigue KM Kennedy KM Head D Williamson A Dahle C Gerstorf D Acker JD Regional brain changes in aging healthy adults: general trends, individual differences and modifiers Cereb Cortex 2005 15 1676 1689 10.1093/cercor/bhi044 15703252

[B21] Oldfield RC The assessment and analysis of handedness: the Edinburgh inventory Neuropsychologia 1971 9 97 113 10.1016/0028-3932(71)90067-4 5146491

[B22] Sled JG Zijdenbos AP Evans AC A nonparametric method for automatic correction of intensity nonuniformity in MRI data IEEE Trans Med Imaging 1998 17 87 97 10.1109/42.668698 9617910

[B23] Collins DL Neelin P Peters TM Evans AC Automatic 3D intersubject registration of MR volumetric data in standardized talairach space J Comput Assist Tomogr 1994 18 192 205 10.1097/00004728-199403000-00005 8126267

[B24] Collins DL Zijdenbos AP Kollokian V Sled JG Kabani NJ Holmes CJ Design and construction of a realistic digital brain phantom IEEE Trans Med Imaging 1998 17 463 468 10.1109/42.712135 9735909

[B25] Collins DL Evans AC ANIMAL: Validation and applications of nonlinear registration-based segmentation Intern J Pattern Recognit Artif Intell 1997 11 1271 1294 10.1142/S0218001497000597

[B26] Pruessner JC Li LM Serles W Pruessner M Collins DL Kabani N Lupien S Evans AC Volumetry of hippocampus and amygdala with high-resolution MRI and threedimensional analysis software: minimizing the discrepancies between laboratories Cereb Cortex 2000 10 433 442 10.1093/cercor/10.4.433 10769253

[B27] Pruessner JC Collins DL Pruessner M Evans AC Age and gender predict volume decline in the anterior and posterior hippocampus in early adulthood J Neurosci 2001 21 194 200 11150336 10.1523/JNEUROSCI.21-01-00194.2001 PMC6762451

[B28] Ifthikharuddin SF Shrier DA Numaguchi Y Tang X Ning R Shibata DK Kurlan R MR volumetric analysis of the human basal ganglia: normative data Acad Radiol 2000 7 627 634 10.1016/S1076-6332(00)80579-6 10952114

[B29] Peterson BS Riddle MA Cohen DJ Katz LD Smith JC Leckman JF Human basal ganglia volume asymmetries on magnetic resonance images Magn Reson Imaging 1993 11 493 498 10.1016/0730-725X(93)90468-S 8316062

[B30] Selemon LD Goldman-Rakic PS Longitudinal topography and interdigitation of corticostriatal projections in the rhesus monkey J Neurosci 1985 5 776 794 2983048 10.1523/JNEUROSCI.05-03-00776.1985 PMC6565017

[B31] Yeterian EH Pandya DN Corticostriatal connections of extrastriate visual areas in rhesus monkeys J Compar Neurol 1995 352 436 457 10.1002/cne.903520309 7706560

[B32] Voss P Collignon O Lassonde M Lepore F Adaptation to sensory loss WIREs Cog Sci 2010 1 308 328 10.1002/wcs.13 26271373

[B33] Voss P Zatorre R Occipital cortical thickness predicts performance on pitch and musical tasks in blind individuals Cereb Cortex 2012 22 2455 2465 10.1093/cercor/bhr311 22095215 PMC4705333

[B34] Konishi K Bohbot VD Spatial navigation strategies correlate with gray matter in the hippocampus of healthy older adults tested in a virtual maze Front Aging Neurosci 2013 5 1 8 23430962 10.3389/fnagi.2013.00001 PMC3576603

